# High resolution melting analysis for the rapid and sensitive detection of mutations in clinical samples: *KRAS *codon 12 and 13 mutations in non-small cell lung cancer

**DOI:** 10.1186/1471-2407-6-295

**Published:** 2006-12-21

**Authors:** Michael Krypuy, Genni M Newnham, David M Thomas, Matthew Conron, Alexander Dobrovic

**Affiliations:** 1 Molecular Pathology Research Laboratory, Peter MacCallum Cancer Centre, Locked Bag 1, A'Beckett St, Melbourne, Victoria 8006, Australia; 2 Centre for Genomics and Predictive Medicine, Peter MacCallum Cancer Centre, Locked Bag 1, A'Beckett St, Melbourne, Victoria 8006, Australia; 3 Department of Pathology, University of Melbourne Department of Medicine, St Vincent's Hospital, Fitzroy 3065, Australia; 4 Department of Respiratory Medicine, St Vincent's Hospital, Fitzroy 3065, Australia; 5 Department of Pathology, University of Melbourne, Parkville, Victoria, 3010, Australia

## Abstract

**Background:**

The development of targeted therapies has created a pressing clinical need for the rapid and robust molecular characterisation of cancers. We describe here the application of high-resolution melting analysis (HRM) to screen for *KRAS *mutations in clinical cancer samples. In non-small cell lung cancer, *KRAS *mutations have been shown to identify a group of patients that do not respond to EGFR targeted therapies and the identification of these mutations is thus clinically important.

**Methods:**

We developed a high-resolution melting (HRM) assay to detect somatic mutations in exon 2, notably codons 12 and 13 of the *KRAS *gene using the intercalating dye SYTO 9. We tested 3 different cell lines with known *KRAS *mutations and then examined the sensitivity of mutation detection with the cell lines using 189 bp and 92 bp amplicons spanning codons 12 and 13. We then screened for *KRAS *mutations in 30 non-small cell lung cancer biopsies that had been previously sequenced for mutations in *EGFR *exons 18–21.

**Results:**

Known *KRAS *mutations in cell lines (A549, HCT116 and RPMI8226) were readily detectable using HRM. The shorter 92 bp amplicon was more sensitive in detecting mutations than the 189 bp amplicon and was able to reliably detect as little as 5–6% of each cell line DNA diluted in normal DNA. Nine of the 30 non-small cell lung cancer biopsies had *KRAS *mutations detected by HRM analysis. The results were confirmed by standard sequencing. Mutations in *KRAS *and *EGFR *were mutually exclusive.

**Conclusion:**

HRM is a sensitive in-tube methodology to screen for mutations in clinical samples. HRM will enable high-throughput screening of gene mutations to allow appropriate therapeutic choices for patients and accelerate research aimed at identifying novel mutations in human cancer.

## Background

With the advent of personalised medicine, there is a compelling need for rapid and accurate methods for detection of nucleic acid sequence changes in clinical specimens. An ideal technology should be sensitive enough to accommodate significant amounts of stromal and normal cell contamination, robust and simple enough to be readily implemented in a diagnostic laboratory, rapid enough to provide important therapeutic information in a clinical timeframe, and cost-efficient. High resolution melting (HRM) is an emerging technique for detection of nucleic acid sequence variation [1] that has enormous potential to meet these clinical demands. We have used detection of codon 12 and 13 mutations in the *KRAS *gene to establish that HRM is a viable methodology that is readily performed both in a research setting and in a routine molecular pathology laboratory.

**Figure 1:**
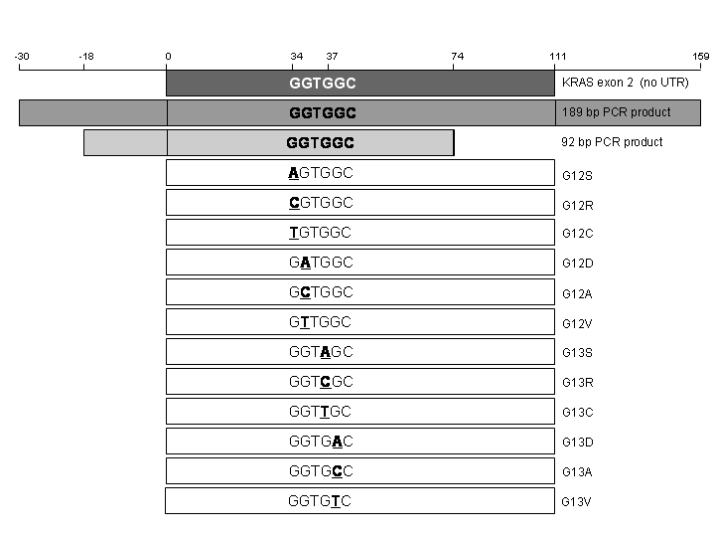
**Location of *KRAS *codon 12 and 13 mutations and PCR amplicons**. Exon 2 of *KRAS *is shown from the ATG without the untranslated region. The position and size of the PCR amplicons used in the HRM assays in relation to exon 2 of *KRAS *is indicated. There are 12 possible single base mutations that can occur at codons 12 and 13 of *KRAS*. All possible mutations are listed along with the corresponding amino acid changes. These mutations are located at positions 64, 65, 67 and 68 of the 189 bp PCR amplicon and at positions 53, 54, 56 and 57 of the 92 bp PCR amplicon.

The ras family genes were originally identified as oncogenes in acutely transforming retroviruses [2]. Three highly homologous ras proteins are encoded by the *KRAS*, *HRAS *and *NRAS *genes. A high frequency of ras mutations has been found in many tumour types. Ras mutations are generally restricted to codons 12 and 13 in exon 2 (Figure 1) and codons 59 and 61 in exon 3, all of which result in constitutive activation of the ras protein [3,4]. Mutated ras proteins have impaired GTPase activity removing the "off switch" thereby resulting in a continual stimulus for cellular proliferation.

While the frequency of mutations and the ras family gene that is mutated varies by cancer type, approximately 30% of all human cancers harbour a mutation in a ras gene with mutations most frequently occurring in *KRAS*. *KRAS *mutations occur in more than 90% of pancreatic adenocarcinomas, in approximately 40% of colorectal cancers and 33% of non- small cell lung carcinomas (NSCLC) [4].

*KRAS *mutations have a mutually exclusive relationship with *EGFR *mutations in NSCLC [5,6]. Mutations in the tyrosine kinase domain of the *EGFR *gene have been observed in NSCLC patients with a response to tyrosine kinase inhibitors such as gefitinib and erlotinib [7-9]. In contrast to *EGFR *mutations, NSCLC patients that have mutations in *KRAS *do not respond to tyrosine kinase inhibitors [10]. NSCLC patients with *EGFR *mutations seem to have a favourable prognosis [11,12] whereas those with *KRAS *mutations have poor prognosis [13-19]. Mutant *KRAS *is thus both a predictive marker and a prognostic marker in NSCLC.

*KRAS *mutation is also a predictive and prognostic marker in other tumour types. In a recent study of metastatic colorectal cancer treated with the monoclonal antibody cetuximab, patients with *KRAS *mutations were resistant to the drug and had a poorer prognosis compared with those without mutations [20]. A review of the literature concluded that *KRAS*, but not p53, mutations, were associated with poorer overall survival in colorectal cancer patients [21]. *KRAS *mutations in pancreatic cancer are also associated with poor prognosis [22-25].

Detection of *KRAS *mutations has often been the primary validation for many of the numerous polymerase chain reaction (PCR) based mutation detection methods. Methodologies described in the literature include single-strand conformation polymorphism (SSCP), denaturing high performance liquid chromatography (DHPLC), denaturant gradient gel electrophoresis, denaturant capillary electrophoresis, automated constant denaturant capillary electrophoresis and temperature denaturant capillary electrophoresis, restriction digestion, allele specific PCR, allele specific nucleotide hybridisation, PCR-reverse dot blot, activated RAS-GTP specific biosensor, oligonucleotide hybridisation assay, mass spectrometry, peptide nucleic acid PCR, and array based techniques [26-42]. All these methods have their advantages and disadvantages in terms of simplicity, performance, sensitivity, turn-around time and cost.

DNA sequencing [43] has been considered the "gold standard" technique because it can identify the specific mutation that may be present. However it has the disadvantages of high relative cost and limited sensitivity. Pyrosequencing has the advantage of greater sensitivity and lower cost [44], but the relatively high cost of the required apparatus has limited its uptake.

In mutation scanning methodologies such as SSCP and DHPLC, samples demonstrating variant profiles are selected for confirmatory analysis with mutation identifying methods such as dideoxy sequencing. An advantage of scanning methodologies is that the amount of sequencing performed is significantly reduced, thereby reducing laboratory costs and analysis time. The range of denaturant capillary electrophoresis methods have many advantages including sensitivity, identification of mutant samples through characteristic peak patterns and high throughput when using a 96-array capillary electrophoresis instrument [30-32]. A disadvantage of the denaturant capillary electrophoresis methods is that access to a dedicated capillary electrophoresis instrument is required.

High resolution melting (HRM) involves the precise monitoring of the change in fluorescence caused by the release of an intercalating DNA dye from a DNA duplex as it is denatured by increasing temperature. Developments in instrumentation and the use of fully saturating intercalating dyes have made HRM analysis possible [1]. An important advantage of HRM over many of the above methods is that it is an in-tube method in which the analysis is performed immediately after the amplification and is thus particularly suitable for medium to high-throughput applications. Methods that require amplified PCR product to be removed from the tube for analysis are inevitably more laborious and require stringent precautions to prevent crossover of PCR products. The HRM methodology has major potential for the rapid and inexpensive detection of DNA sequence variations such as polymorphisms and germline mutations in DNA [45,46].

A particularly desirable HRM application is the detection of cancer specific mutations in tumour biopsies. HRM has been used to identify somatic mutations in the *c-KIT*, *BRAF*, *HER2 *and *EGFR *genes [47-53]. In this study, we set out to develop a sensitive, clinically useful HRM assay to detect somatic mutations in the *KRAS *gene. We validated the assay with cell lines with known *KRAS *mutations, and with a panel of NSCLC biopsies that were also tested for *KRAS *mutations by sequencing.

## Methods

### Samples and DNA extraction

Thirty NSCLC tumours were analysed, including 22 adenocarcinomas and 8 large cell carcinomas. Patients consented for the use of their tissue through the "Molecular profiling of early stage non-small cell lung cancer to predict clinical course and identify pathogenically important genes" study of St. Vincent's Hospital or though the Peter MacCallum Cancer Centre Tissue Bank. The samples were reviewed by an experienced pathologist. DNA was extracted from the lung cancer biopsies using the DNeasy Tissue kit (Qiagen, Hilden, Germany) according to the manufacturer's protocol or by phenol-chloroform extraction. Wild-type control DNA and cell line DNA were extracted using a salting out method [54]. The 22 adenocarcinoma samples were previously sequenced for mutations in *EGFR *exons 18–21.

### Assay design and PCR conditions

Primers were designed to span codons 12 and 13 of the *KRAS *gene (see Figure 1). Primers for the 189 base pair (bp) amplicon which spanned all of exon 2 were 5'-TCATTATTTTTATTATAAGGCCTGCTGAA-3' (forward) and 5'-CAAAGACTGGTCCTGCACCAGTA-3' (reverse). Primers for the shorter 92 bp amplicon were 5'-ttataagGCCTGCTGAAAATGACTGAA-3' (forward) and 5'-TGAATTAGCTGTATCGTCAAGGCACT-3' (reverse). The intercalating dye used was SYTO 9 (Invitrogen, Carlsbad, USA). The reaction mixture was made up using HotStarTaq (Qiagen) and consisted of 10 ng of genomic DNA, 1× PCR buffer, 2.5 mM MgCl_2 _total, 200 nM of each primer, 200 μM of dNTPs, 5 μM of SYTO 9, 0.5U of HotStarTaq polymerase and PCR grade water in a volume of 20 μL. All PCR reactions were performed in duplicate.

PCR cycling and HRM analysis was performed on the Rotor-Gene 6000™ (Corbett Research, Mortlake, New South Wales, Australia). The 189 bp amplicon was run according to the following conditions; one cycle of 95°C for 15 minutes; 40 cycles of 95°C for 15 seconds, 60.7°C for 15 seconds, 72°C for 15 seconds; one cycle of 95°C for 1 second, 72°C for 90 seconds and a melt from 72 to 95°C rising at 0.2°C per second. The 92 bp amplicon was run according to the following conditions; one cycle of 95°C for 15 minutes; 40 cycles of 95°C for 15 seconds, 67.5°C for 15 seconds, 72°C for 15 seconds; one cycle of 95°C for 1 sec; and a melt from 72 to 95°C rising at 0.2°C per second.

### High resolution melting analytical sensitivity testing

For information about the cell lines used in this study see Table 1. The HCT116 cell line was mixed with wild-type DNA in dilutions of 50%, 25%, 10%, 5%, 1%. The A549 and RPMI8226 cell lines were mixed with wild-type DNA in serial dilutions of 50%, 25%. 12%, 6% and 3%. All dilutions were tested using both the 189 bp and 92 bp amplicons.

### DNA sequencing

After HRM analysis, the PCR products were column purified using the PCR-M clean up kit (Viogene, Taipei, Taiwan) according to the manufacturer's instructions. The PCR products were eluted in a 30 μl volume, and 6 μl was treated with ExoSapIT (GE Healthcare, Buckinghamshire, England) according to the manufacturer's instructions. The purified PCR product was then used as template in cycle sequencing with the Big Dye Terminator v3.1 kit (Applied Biosystems, Foster City, CA). The reaction mix consisted of 1× terminator premix, 1× sequencing buffer, 667 nM primer and 3.5 μl of cleaned template in a 15 μl total volume. The reactions were run on a GeneAmp 9700 thermocycler (Applied Biosystems) according to the following protocol; one cycle of 95°C for 15 minutes; 25 cycles of 95°C for 10 seconds, 55°C for 5 seconds, 72°C for 4 minutes. The sequencing reactions were ethanol precipitated and run on a 3100 Genetic Analyser (Applied Biosystems,). Sequencing data was analysed using Sequencher™ 4.6 (Gene Codes Corporation, Ann Arbor, MI).

## Results

### Assay validation and sensitivity testing

**Figure 2:**
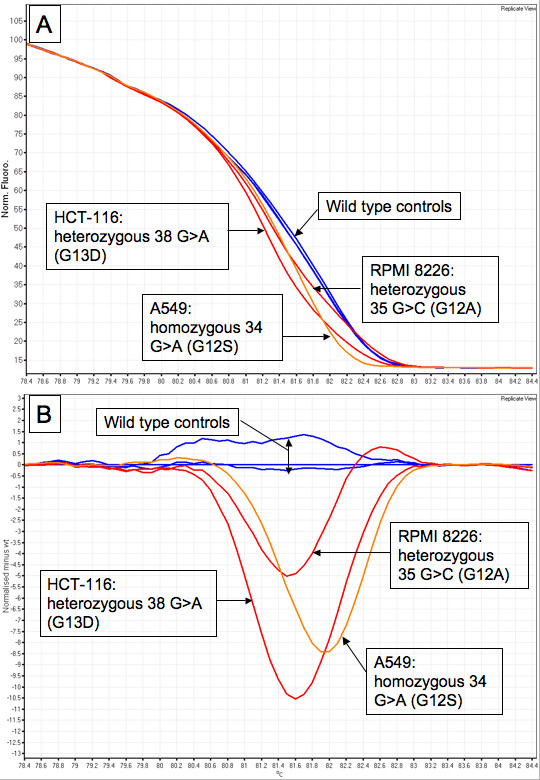
**Difference plots of *KRAS *mutated cell lines**. **Panel A: **A normal plot of the wild-type controls with the cell line samples. Fluorescence is on the Y-axis and temperature in degrees Celsius is on the X-axis. **Panel B: **A difference plot of the wild-type controls with the cell line samples. All cell line samples were compared to the median wild-type control sample to produce the plot.

Cancer cell lines with known *KRAS *mutations (Table 1) were first used to test the HRM methodology. HRM was able to discriminate between wild-type DNA and the different mutations present in the cell line DNAs using the 189 bp amplicon flanking exon 2. Figure 2 shows a normalised plot and a difference plot of the HRM data using wild-type DNA as a baseline. The plasmacytoma cell line RPMI8226 has a heterozygous 35G > C (G12A) *KRAS *mutation and the colon cancer cell line HCT116 has a heterozygous 38G > A (G13D) mutation. Both these cell lines showed typical heteroduplex melting patterns and were readily distinguishable from wild-type samples. The lung cancer line A549 has a homozygous 34G > A (G12S) mutation and, as expected, it produced a curve that was similar in shape to the wild-type but with earlier melting of the amplified product compared to wild-type samples, which is consistent with the lower thermal stability of AT base pairs relative to GC base pairs.

Primers giving a 92 bp PCR amplicon were designed to test the effect of amplicon size on the sensitivity of detecting mutant sequence in a background of normal DNA. The sensitivity of mutation detection using the 92 bp and 189 bp PCR amplicons was tested by assaying a range of dilutions of the cell line DNAs in normal DNAs.

**Figure 3:**
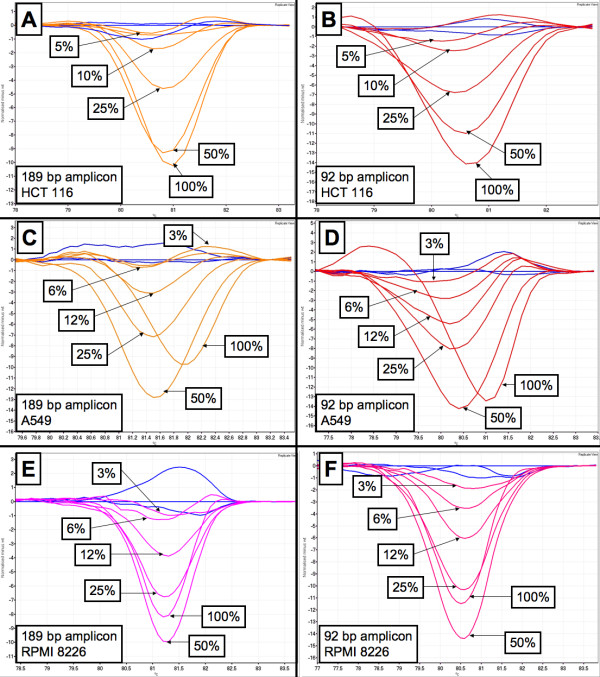
**Sensitivity of the *KRAS *HRM assay for different amplicons**. **Panel A**: The HCT116 dilutions were compared to the median wild-type control sample to produce the difference plot for the 189 bp amplicon. **Panel B: **The HCT116 dilutions were compared to the median wild-type control sample to produce the difference plot for the 92 bp amplicon. **Panel C: **The A549 dilutions were compared to the median wild-type control sample to produce the difference plot for the 189 bp amplicon. **Panel D: **The A549 dilutions were compared to the median wild-type control sample to produce the difference plot for the 92 bp amplicon. **Panel E: **The RPMI8226 dilutions were compared to the median wild-type control sample to produce the difference plot for the 189 bp amplicon. **Panel F: **The RPMI8226 dilutions were compared to the median wild-type control sample to produce the difference plot for the 92 bp amplicon.

HCT116 DNA was mixed with wild-type DNA in proportions of 50%, 25%, 10%, 5% and 1%. The A549 and RPMI8226 cell lines were mixed with wild-type DNA in dilutions of 50%, 25%, 12%, 6%, and 3%. Figure 3 shows the difference plots for both PCR amplicons with the 3 cell line dilutions. The cell line dilutions were assessed by several individuals in a blinded fashion where scoring depended on being able to confidently differentiate the mutant containing DNA dilutions from the normal DNA samples. In all cases there was an increase in sensitivity with the shorter 92 bp amplicon. Using the 189 bp amplicon, we were able to detect 10% HCT116 in wild-type DNA whereas using the 92 bp amplicon, we were able to detect 5% HCT116 DNA. A549 and RPMI8226 gave essentially identical results: 12% cell line DNA was detectable with the 189 bp amplicon whereas 6% cell line DNA was detected using the shorter 92 bp amplicon.

### KRAS mutation detection in NSCLC samples

The 189 bp and 92 bp PCR amplicons were used to screen for mutations in a panel of DNAs derived from biopsies of 22 adenocarcinomas and 8 large cell carcinomas. We detected the presence of nine aberrant curves in the 30 samples assayed. The nine mutations identified by HRM using the 92 bp amplicon were also detected using 189 bp amplicon. Following HRM analysis, the 189 bp PCR amplicons from each sample were sequenced. The sequencing results confirmed the results of the HRM analysis.

**Figure 4:**
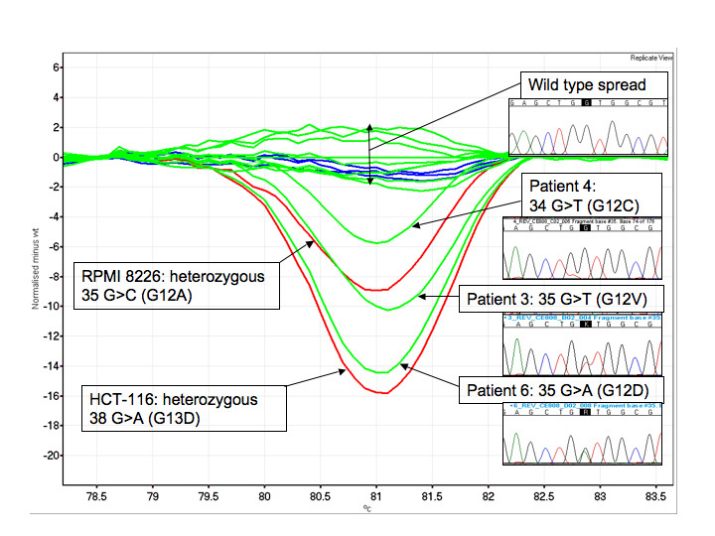
**Difference plot and sequencing traces of various mutations in *KRAS***. Sequencing plots for each sample are below the patient numbers. The samples were compared to the median wild-type control sample to produce the difference plot. The sequencing traces for patients 3 and 6 showed the presence of a 35G > T and 35G > A mutations respectively.

Figure 4 shows a difference plot and sequencing traces for cell line controls, wild-type samples and a selection of patients with *KRAS *mutations. Patients 3 and 6, which had HRM curves that deviated greatly from the wild-type curves, showed the presence of a 35G > T and 35G > A mutations respectively with prominent mutant peaks. Patient 4 had a HRM curve that deviated less from the wild-type plot and sequencing showed a 34G > T mutation with a relatively small peak for the mutant T base. The summary of all mutations detected in the panel of 30 lung cancer biopsies is presented in Table 2.

## Discussion

A wide range of mutation detection methodologies exist, of which sequencing has been considered the gold standard because of its ability to identify the specific DNA sequence change that has occurred. However, dideoxy sequencing is rarely sensitive below a 10% mutant allele frequency, which corresponds to a threshold of 20% tumour cells heterozygous for a mutation [44]. The sensitivity of sequencing can be increased by microdissection (ranging from relatively crude scraping to laser capture) to enrich for tumour cells, but this adds to the complexity of genetic testing. The variety of methodologies used for mutation detection probably account for much of the wide divergence of mutation frequencies often reported for particular genes in given cancer types.

High resolution melting (HRM) methodology represents a significant advance for mutation detection in tumour specimens. The developments in intercalating dye technology have played a major role in the emergence of HRM methodology. Different base substitutions produce slight differences in melting behaviour and the resolution of these melting differences requires an appropriate intercalating dye. SYBR green, the most widely used intercalating dye for monitoring PCR, is not suitable for HRM analysis because it can only be used at non-saturating concentrations. Thus, SYBR green molecules that dissociate from DNA during melting can re-intercalate into regions of un-melted double stranded DNA [55]. This phenomenon of dye redistribution can mask the small differences in melting behaviour. New generation intercalating dyes such as LC Green and LC Green PLUS allow accurate genotyping of single-nucleotide polymorphisms and sensitive mutation scanning [1,56]. Monis et al. (2005) showed that SYTO 9 was suitable for fluorescence monitoring of PCR and standard melting analysis [57]. In this study, we have shown that SYTO 9 is a suitable dye for mutation scanning with HRM, as it was able to sensitively detect *KRAS *mutations in cell line controls and in tumour samples.

An advantage of performing HRM analysis on a real time PCR machine with HRM capability (for which this is the first report), is that the PCR amplification and HRM analysis are performed in the one run and the results are available for analysis at the end of the run. This allows assessment of amplification for all samples before HRM analysis as a quality control measure. In our experience, samples with poor or late amplification must be treated with some caution in HRM analysis. Use of a single instrument also minimises the amount of manual handling which improves turnaround times.

We observed a degree of variation of the melting plots within wild-type samples, which imposed a lower limit on the sensitivity of mutation detection with the tumour sample set. The presence of small amounts of non-specific amplicons, differing salt or inhibitor concentrations, or differences in PCR amplification between samples may contribute to the variation. It is clear that as the spread of wild-type curves increases, the sensitivity of mutation detection decreases. Thus instrumentation with greater temperature uniformity across the samples assayed may have an advantage in mutation scanning.

Difference plots are visually the best way to compare melting profiles. However, difference plots emphasise small variations in the melting profiles between different samples giving rise to a wild-type spread. Nevertheless, wild-type sample plots were quite distinct from samples with mutations. We chose a sample from the centre of the distribution of the wild-type samples as the normalisation sample for the construction of the difference plots to standardise the interpretation. The presence of mutated sequence, even in low proportion, produced difference plots that were readily discriminated from wild-type samples as seen in the sensitivity testing of the assay (Figure 3).

In a study that used HRM to screen for mutations in the *RET *proto-oncogene, it was found that some mutations could be directly genotyped from the melting curve shapes [58]. However, the applicability of shape analysis in tumour samples will be compromised by the influence on the shape of the melting profile of varying degrees of heteroduplex formation due to the proportion of normal DNA present in the tumour biopsy. Determination of the precise mutation will depend on more complex methods such as allele-specific competitive blocker PCR, dideoxy sequencing or pyrosequencing.

Sensitivity testing is an essential step in the development of HRM assays for mutation detection and different sensitivities may be achieved with certain mutations or as in this report, with particular PCR amplicons. Liew et al. previously examined the role of the size of the PCR product in SNP genotyping and found that smaller amplicons gave better differentiation between genotypes [59]. Consistent with those results, we found that use of a smaller amplicon increased the sensitivity of mutation detection for KRAS mutations in the cell lines tested. The 189 bp and 92 bp PCR amplicons have a G+C content of 37% and 43% respectively and this may also have played a role in the altered sensitivity of mutation resolution.

Identifying mutations will depend on being able to confidently differentiate the aberrant curves from the wild-type samples. The cell line dilution results were assessed by several independent observers in a blinded fashion and the results indicate that 5–6% tumour cell line DNA in a background of normal DNA could be detected using the 92 bp PCR amplicon. This will allow confident screening for mutations in samples that have at least 10% tumour cells.

The results of our cell line dilution studies indicate that in some cases, mutations detected by HRM will not be detectable by dideoxy sequencing. These situations must be considered when setting up mutation detection algorithms. Pyrosequencing may be the confirmatory sequencing method of choice as it has been shown to be sensitive to 5% mutant sequence [44] and minimises the amount of reactions needed compared to allele-specific PCR methods. We did not observe a discrepancy between HRM and dideoxy sequencing in our study. This is likely to be due to the pathological review and dissection of areas consisting of at least 50% tumour cells. This argues for a testing algorithm consisting of a pathological review to mark out sections of interest and a crude microdissection to purify the tumour component.

In the case of KRAS codon 12 and 13 mutations, allele-specific competitive blocker PCR may be a cost-effective sensitive method to identify mutations detected by HRM which has the ability to identify low level mutations identified by HRM not detectable by dideoxy sequencing [60]. Allele-specific competitive blocker PCR has been shown to be sensitive down to 0.1% mutant sequence in a background of normal DNA [61].

Somatic mutations of *KRAS *occurring at bases 34, 35, 37 and 38 from the start codon give rise to amino acid substitutions at codon 12 and 13. We observed six of the possible 12 mutations, five of which were seen in the patient specimens. The G12S (35G > A) and G12R (35G > C) mutations, which were not observed in our relatively small patient set, are the rarest codon 12 mutations in lung cancer [62]. G13D was the only codon 13 mutation observed but all of the codon 13 mutations are rare with only G13C (37G > T) and G13D (38G > A) occurring at any appreciable frequency. All of the samples were sequenced and no other mutations were detected that had not been detected by HRM. It might be considered that a limitation of this study is that all possible *KRAS *mutations were not tested. However, similar sensitivities would be achieved for the other mutations because the lower melting temperature of heteroduplexes is the basis of the sensitivity of mutation detection using HRM.

The frequency of *KRAS *mutations in this small sample set was higher than many reports in the literature. There were 7 mutations in 22 (32%) in the adenocarcinomas and 2 mutations in 8 (25%) large cell carcinomas but these frequencies may change substantially when larger numbers of specimens are analysed. As previously observed by others [5,6], there was a mutually exclusive relationship between *KRAS *and *EGFR *mutation status for our sample set. The adenocarcinoma samples that had *EGFR *mutations did not present with *KRAS *mutations. NSCLC patients with mutated *KRAS *do not respond to tyrosine kinase inhibitors and have a poorer prognosis than patients without mutations [10,13-19]. Early identification of these patients will allow appropriate and cost-effective treatment decisions to be made by clinicians.

## Conclusion

In conclusion, we have presented a robust assay for screening for *KRAS *codon 12 and 13 mutations that is applicable to clinical samples. The main limitation of HRM is that the precise mutation cannot be readily identified and it thus needs to be used in conjunction with a sequencing method. Nevertheless, because HRM is both inexpensive and high throughput, we believe that HRM is now the method of choice for screening tumour samples for somatic mutations prior to sequencing, especially when the mutations are likely to occur at low frequency. As targeted therapies focus increasingly on key mutations in oncogenic pathways, it will be necessary to screen not only for multiple exons within candidate genes, but also multiple genes within key pathways to confirm or exclude involvement of that pathway. The rate limiting step in genetic analysis of tumours is now the collection of samples and the preparation of the DNA.

## Authors' contributions

MK carried out the molecular genetic studies, data analysis and wrote the manuscript. AD conceived the study, designed the primers, participated in the study design and coordination and co-wrote the manuscript. GN, MC, DT were responsible for human research ethic committee submissions to acquire the samples used in the study, their storage and processing, participated in the study design and coordination and contributed their ideas to the manuscript. All authors read and approved the final manuscript.

## Pre-publication history

The pre-publication history for this paper can be accessed here:



## Figures and Tables

**Table 1:** Cell line controls with mutations in *KRAS*

**Cell line**	**Tissue**	**Mutation**	**Genotype**	**Amino acid**
A549	lung carcinoma	34G > A	homozygous	G12S
RPMI8226	plasma cell myeloma	35G > C	heterozygous	G12A
HCT116	colon carcinoma	38G > A	heterozygous	G13D

**Table 2:** Summary of results for 30 lung cancers screened for mutations in *KRAS*

**Patient**	**Histology**	**HRM**	**Sequencing**	**AA change**	***EGFR *sequencing**
1	Adenocarcinoma	wt	wt	-	wt
2	Adenocarcinoma	wt	wt	-	wt
3	Adenocarcinoma	mut	35G > T	G12V	wt
4	Adenocarcinoma	mut	34G > T	G12C	wt
5	Adenocarcinoma	wt	wt	-	wt
6	Adenocarcinoma	mut	35G > A	G12D	wt
7	Adenocarcinoma	wt	wt	-	wt
8	Adenocarcinoma	wt	wt	-	wt
9	Adenocarcinoma	wt	wt	-	exon 19 del
10	Adenocarcinoma	wt	wt	-	exon 19 del
11	Adenocarcinoma	wt	wt	-	wt
12	Adenocarcinoma	wt	wt	-	wt
13	Adenocarcinoma	mut	35G > T	G12V	wt
14	Adenocarcinoma	mut	34G > T	G12C	wt
15	Adenocarcinoma	wt	wt	-	wt
16	Adenocarcinoma	wt	wt	-	wt
17	Adenocarcinoma	wt	wt	-	exon 19 del
18	Adenocarcinoma	mut	38G > A	G13D	wt
19	Adenocarcinoma	wt	wt	-	wt
20	Adenocarcinoma	wt	wt	-	wt
21	Adenocarcinoma	wt	wt	-	wt
22	Adenocarcinoma	mut	35G > T	G12V	wt
23	Large cell	wt	wt	-	Not done
24	Large cell	wt	wt	-	Not done
25	Large cell	mut	35G > C	G12A	Not done
26	Large cell	wt	wt	-	Not done
27	Large cell	wt	wt	-	Not done
28	Large cell	wt	wt	-	Not done
29	Large cell	mut	35G > T	G12V	Not done
30	Large cell	wt	wt	-	Not done

HRM – high resolution melting, wt – wild-type, mut – mutant, AA change – amino acid change, exon 19 del – EGFR Exon 19 deletion
